# Mapping of key performance indicators for palliative care in intensive care settings: A scoping review protocol

**DOI:** 10.12688/hrbopenres.14430.1

**Published:** 2026-04-30

**Authors:** Yvonne King, Peter May, Anne-Marie Brady

**Affiliations:** 1 Trinity Centre for Practice & Healthcare Innovation,School of Nursing and Midwifery, Trinity College Dublin,The University of Dublin, Dublin, Leinster, Ireland; 2 Cicely Saunders Institute of Palliative Care Policy & Rehabilitation,Florence Nightingale Faculty of Nursing Midwifery & Palliative Care, King's College London, London, England, UK; 3 School of Medicine, Trinity College Dublin, Trinity College Dublin, Dublin, Leinster, Ireland

**Keywords:** Critical Care; End-of-Life Care; Intensive Care Units; Key Performance Indicators, Palliative Care; Quality Indicators, Scoping Reviews

## Abstract

**Background:**

Palliative care integration in intensive care units (ICUs) is becoming increasingly important in terms of patient-centred care delivery. While both generalist (primary) palliative care provided at the ICU level and specialist palliative care (SPC) services support delivery, there is limited clarity regarding how such integration and care can be measured. Key performance indicators are generally identified as indicators of the quality of healthcare. However, relevant palliative care key performance indicators for the ICU are poorly defined and vary across policies, regulations, and the literature.

**Aim:**

To identify and map key performance indicators to inform a domain-based framework for evaluating palliative care delivery in ICUs.

**Methods:**

This study will be conducted as a scoping review following the PRISMA-ScR guidelines. We will undertake a systematic search of peer-reviewed records, grey literature, national health system sources, regulatory bodies, and professional organisations. Once extracted, we will analyse the key performance indicators and group them into domains reflecting both generalist and specialist palliative care delivery.

**Conclusions:**

This will result in a comprehensive synthesis of key performance indicators and inform the development of a structured framework to support the evaluation and implementation of palliative care delivery in the ICU.

## Introduction

Integrating palliative care into intensive care units (ICU) is a growing priority in international policy and clinical guidance ( Centre of Advanced Palliative Care [CAPC], 2019; Kesecioglu et al. 2018; World Health Organisation [WHO], 2014). Patients in the ICU are older and face a high symptom burden, complex decisions, and uncertain outcomes, requiring clinicians to provide generalist palliative care and seek specialist support as needed ( Akinosoglou et al., 2023; Giabicani et al. 2023; Hua et al. 2014). Evidence indicates that early palliative care, integrated alongside active treatment beyond end-of-life care, facilitates timely identification of patient needs, enhances decision-making and quality of patient centred care ( Araujo et al., 2023; Aslakson et al., 2014; Ma et al., 2019) **.**

Despite this, there is no standardised way to measure the quality or effectiveness of palliative care integration or delivery in ICU settings. Key performance indicators (KPIs) are widely used across healthcare to support quality improvement, benchmarking, and accountability ( WHO, 2018). However, indicators relevant to ICU palliative care remain varied across policy documents, regulatory standards, and academic literature, resulting in limited synthesis and little conceptual clarity, limiting their application in practice, quality improvement, and system-level evaluation.

This study builds on our previous work, which mapped key palliative care characteristics of ICU integration models, by translating these into a structured framework of measurable indicators to support system-level evaluation ( King Y. et al. 2026). It supports national research priorities in Ireland by outlining how palliative care delivery can be measured and evaluated for individuals with life-limiting illness ( All Ireland Institute of Hospice and Palliative Care [AIIHPC], 2025). A scoping review is appropriate for identifying and mapping this diverse evidence base ( Peters et al., 2020). These findings will inform the development of an evidence-based KPI framework to support quality improvement, benchmarking, and accountability in ICU palliative care.

## Protocol

**Study design:** This scoping review will be conducted in accordance with the methodological framework proposed by Arksey and O’Malley (2005), with additional guidance from the Joanna Briggs Institute, and will be reported in line with the PRISMA-ScR guidelines.

**Aim**: To identify and map key performance indicators (KPIs) to inform a framework for evaluating palliative care delivery in intensive care units (ICUs).

### Objectives

1. To identify KPIs used to evaluate palliative care delivery in ICUs.
2. To map the domains and categories of these indicators.
3. To examine how these indicators are defined and measured across studies.
4. To inform the development of a structured framework that organises domains and KPIs for evaluating palliative care delivery in the ICU

Table 1 details the Population, Concept, and Context (PCC).

**Table 1.** Population, Concept, and Context (PCC) framework guiding the review.

Population	Concept	Context
Adult patients in ICU	KPIs, quality measures & outcome measures	Delivery & integration of palliative care in ICU settings

* ICU = Intensive Care Unit; KPIs = Key Performance Indicators.

**Search strategy:** We will conduct a comprehensive search using controlled vocabulary (e.g., MeSH terms) and free-text terms derived from the PCC framework. The search will focus on three core concepts: palliative care, intensive care, and key performance or quality indicators (see Table 2). The full search string for all databases is available on the Open Science Framework (OSF) at 10.17605/OSF.IO/W5J8V We will combine synonymous terms using OR operator within each key concept to maximise search sensitivity. Core concepts will then be combined using the AND operator. Truncation and phrase searching will capture variations within terminology. There is no single index term for key performance indicators, so we will use a mix of subject headings and free-text terms. These will include quality indicators, outcomes, audit, benchmarking, and improvement. We will search MEDLINE, CINAHL, and Embase. See Table 3 for full details. Initially, we will search all years, then apply a date limit from 2014 onward. The date limit is justified by the shift towards integrated palliative care in contemporary acute healthcare policy. This builds on earlier WHO guidance that positioned palliative care alongside active treatment ( WHO, 2002; WHO & Worldwide Hospice Palliative Care Alliance, 2014). We will identify relevant indicators by systematically searching the named databases and grey literature from national health systems, regulatory bodies, and professional organisations. This search process will be documented and reported using a PRISMA-ScR flow diagram.

**Table 2.** Key concepts used to inform the search strategy.

Concept	Content
#1	Palliative care
#2	ICU/critical care
#3	KPIs/quality indicators

* ICU = Intensive Care Unit; KPIs = Key Performance Indicators.

**Table 3.** Information sources to be searched, including databases and grey literature sources.

Source type	Sources
**Bibliographic databases**	MEDLINE, CINAHL, Embase
**International organisations**	World Health Organisation (WHO), European Association for Palliative Care (EAPC)
**Policy and guideline bodies**	National Institute for Health and Care Excellence (NICE), Health Information and Quality Authority (HIQA), Department of Health (Ireland)
**National health systems**	Health Service Executive (HSE, Ireland)
**Professional organisations**	All Ireland Institute of Hospice and Palliative Care (AIIHPC); Intensive Care Society (UK & Ireland); Society of Intensive Care Medicine (Ireland); American Thoracic Society (USA); Australian and New Zealand Intensive Care Society (ANZICS)
**Research and policy groups**	Center to Advance Palliative Care (CAPC), Robert Wood Johnson Foundation workgroups
**Clinical programmes**	National Clinical Programme for Palliative Care (Ireland)

**Eligibility criteria:** We developed the eligibility criteria using the PCC structure to guide study selection. This strategy will include studies and documents relating to adult patients (≥18 years) receiving care in ICU settings, also known as critical care settings. We plan to exclude studies focusing on paediatric populations. This review focuses on key performance indicators (KPIs), quality indicators, and outcome measures relevant to palliative care, within adult ICUs. This includes indicators relating to quality of care, service delivery, clinical processes, patient or family outcomes, and audit and benchmarking. Applying limits to the last 12 years ensure relevancy to today’s practice.

**Study selection:** Titles and abstracts will be screened against inclusion criteria, followed by full-text review. Table 4 shows the inclusion/exclusion criteria. Screening will be conducted by two reviewers with discrepancies resolved through discussion with a third arbitrator.

**Table 4.** Inclusion and exclusion criteria for study selection.

Inclusion	Exclusion
Studies and documents describing: • KPIs • quality indicators • outcome measures Related to: • palliative care • ICU/critical care Sources: • peer-reviewed literature • national policy • regulatory frameworks • audit reports • grey literature	• Non-ICU settings • Paediatric ICU settings only • Opinion pieces • Abstracts • Non-peer reviews • >10 years old • Publications where “palliative care” is not explicitly mentioned

* ICU = Intensive Care Unit

**Data extraction & charting:** We will extract data using a standardised charting form, detailed in Table 5. Planned extraction data include study characteristics (authors, year of publication, country/region, and study design) and ICU type (e.g., medical, surgical, or mixed intensive care units). Planned extracted data include the type of palliative care delivered (e.g., generalist or specialist modes) and the domains of care (physical, psychological, social, spiritual, and end-of-life). For each identified KPI, we will record name, definition, type (structure, process, or outcome), measurement methods, data sources, and the timing of measurement during an ICU stay.

**Table 5.** Data extraction variables for charting included studies.

Category	Variable	Description/Purpose
**Study Characteristics**	Author(s)	First author and year of publication
	Year of publication (2014-present)	To ensure clinical and policy relevance
	Country/region	Geographic context of study
	Study design	e.g. qualitative, quantitative, mixed-methods, policy document
	ICU setting	Type of ICU (e.g. medical, surgical, mixed, specialist ICU)
**Concept**	Type of palliative care delivery	Generalist vs specialist palliative care; model of integration within ICU
	Domain of care	Physical, psychological, social, spiritual, end-of-life care
**Indicators/KPIs**	Indicator name	Name/label of KPI or quality indicator
	Indicator definition	How the indicator is defined
	Indicator type	Structure, process, or outcome measure
	Measurement method	How the indicator is measured (e.g. audit, tool, scale)
	Data source	e.g. patient records, ICU databases, administrative data
	Timing of measurement	When indicator is assessed (e.g. ICU admission, during stay, end-of-life)
**Implementation/Use**	Purpose of indicator	Quality improvement, benchmarking, audit, policy
	Level of application	Patient-level, ICU/unit-level, system-level
	Feasibility/usability	Reported facilitators or challenges to use in ICU
**Key Findings**	Main findings	Summary of relevant results
	Recommendations	Author recommendations for ICU palliative care practice/policy
**Relevance to Review**	Relevant to framework development	Role in informing palliative care ICU KPI framework

* ICU = Intensive Care Unit; KPIs = Key Performance Indicators

We also plan to extract the purpose and application of the identified KPIs. (e.g., quality improvement, benchmarking, audit), the level of implementation (patient, unit, or system level), and any documented facilitators or challenges to them. Finally, key findings and recommendations relevant to ICU palliative care will be documented, including their relevance to the development of a KPI framework. This data will support the interpretation of the relevance, applicability, and feasibility of the identified KPIs and inform the identification of meaningful, implementable indicators for inclusion in the proposed KPI framework. We plan to pilot a sample of included studies and refine this chart iteratively to ensure consistency and relevance. Data will be extracted by one reviewer and independently checked for accuracy by a second reviewer.

**Data synthesis:** Extracted KPIs will be analysed using an, inductive iterative thematic analysis approach to identify key domains and patterns ( Braun & Clarke, 2021). Indicators will be grouped into domains reflecting core components of palliative care delivery and integration within ICU settings.

We plan to capture both generalist (primary) palliative care and specialist palliative care concepts, indicators and domains within ICU practice. 

**Results**: We will provide a descriptive summary of the identified KPIs, including their categorisation into domains and themes. The findings will be mapped against the review objectives to provide an overview of the types of indicators used to evaluate palliative care delivery in ICUs. We will develop a conceptual framework that organises identified domains and KPIs, and classify KPIs according to the Donabedian Model (structure, process, and outcomes) to support system-level evaluation.

Potential limitations of this scoping review will be acknowledged, including restrictions on study design, reporting quality, and the absence of a formal quality appraisal. As this review aims to map KPIs for evaluating palliative care delivery within the ICU, we will include studies regardless of methodological design or quality to ensure comprehensive coverage. This is consistent with guidance from the Joanna Briggs Institute and others, which emphasises that critical appraisal in scoping reviews should be guided by the review objectives ( Arksey & O’Malley, 2005; Peters et al., 2020). Limitations related to the breadth of the search and potential gaps in the available evidence will also be considered.

The findings of this review will inform the development of a framework for evaluating palliative care delivery in ICUs and identify gaps in the existing evidence base to guide future research and practice.

## Conclusion

This scoping review will map available evidence and policies on KPIs for ICU palliative care. It will clarify these PC KPIs for ICU care and their application. The review will address an important gap in the literature and support the development of an evidence-based KPI framework. These findings are expected to inform clinical practice, quality improvement initiatives, and policy development, enhancing the integration and evaluation of palliative care in ICU settings.

## Patient and public involvement

Patients and the public were not involved in the design of this protocol.

## Ethics and consent

Ethical approval is not required as the study involves analysis of publicly available data.

## Dissemination

Findings will be disseminated through peer-reviewed publication, conference presentations, and engagement with policy and clinical stakeholders. The resulting framework aims to support evaluation and implementation of integrated palliative care within ICU settings.

**Registration**: The protocol has been registered on the Open Science Framework (OSF) ( https://osf.io/w5j8v/).

**Study status:** The study is currently at the development stage.

## Data Availability

No data associated with this article. Open Science Framework (OSF). https://doi.org/10.17605/OSF.IO/W5J8V ( King Y. et al. 2026). Relevant study materials (e.g., collection tools, and supplementary documentation) will be developed during the study and deposited in this file. This project contains the following underlying data:
• Search string • PRISMA-ScR Checklist. Data is available under the terms of the CC-By Attribution 4.0 International.
