# Mapping of key performance indicators for palliative care in intensive care settings: A scoping review protocol

**DOI:** 10.12688/hrbopenres.14430.2

**Published:** 2026-08-08

**Authors:** Yvonne King, Peter May, Anne-Marie Brady

**Affiliations:** 1 Trinity Centre for Practice & Healthcare Innovation,School of Nursing and Midwifery, Trinity College Dublin,The University of Dublin, Dublin, Leinster, Ireland; 2 Cicely Saunders Institute of Palliative Care Policy & Rehabilitation,Florence Nightingale Faculty of Nursing Midwifery & Palliative Care, King's College London, London, England, UK; 3 School of Medicine, Trinity College Dublin, Trinity College Dublin, Dublin, Leinster, Ireland

**Keywords:** Critical Care; End-of-Life Care; Intensive Care Units; Key Performance Indicators, Palliative Care; Quality Indicators, Scoping Reviews

## Abstract

**Background:**

Palliative care integration in intensive care units (ICUs) is becoming increasingly important in terms of patient-centred care delivery. While both generalist (primary) palliative care provided at the ICU level and specialist palliative care (SPC) services support delivery, there is limited clarity regarding how such integration and care can be measured. Key performance indicators are generally identified as indicators of the quality of healthcare. However, relevant palliative care key performance indicators for the ICU are poorly defined and vary across policies, regulations and the literature.

**Aim:**

To identify and map key performance indicators to inform a domain-based framework for evaluating palliative care delivery in ICUs.

**Methods:**

This study will be conducted as a scoping review following the PRISMA-ScR guidelines. We will undertake a systematic search of peer-reviewed records, grey literature, national health system sources, regulatory bodies, and professional organisations. Once extracted, we will analyse the key performance indicators and group them into domains reflecting both generalist and specialist palliative care delivery.

**Conclusions:**

This will result in a comprehensive synthesis of key performance indicators and inform the development of a structured framework to support the evaluation and implementation of palliative care delivery in the ICU.

## Introduction

Integrating palliative care into intensive care units (ICU) is a growing priority in international policy and clinical guidance ( Centre to Advanced Palliative Care [CAPC], 2019; Kesecioglu et al., 2018; World Health Organisation [WHO], 2014). Patients in the ICU are older and face a high symptom burden, complex decisions, and uncertain outcomes, requiring clinicians to provide generalist palliative care and seek specialist support as needed ( Akinosoglou et al., 2023; Giabicani et al., 2023; Hua et al., 2014). Evidence indicates that early palliative care, integrated alongside active treatment beyond end-of-life care, facilitates timely identification of patient needs, enhances decision-making and quality of patient centred care ( Araujo et al., 2023; Aslakson et al., 2014; Ma et al., 2019).

Despite this, there is no standardised way to measure the quality or effectiveness of palliative care integration or delivery in ICU settings. Key performance indicators (KPIs) are widely used across healthcare to support quality improvement, benchmarking, and accountability ( WHO, 2018). However, indicators relevant to ICU palliative care remain varied across policy documents, regulatory standards, and academic literature, resulting in limited synthesis and little conceptual clarity, limiting their application in practice, quality improvement, and system-level evaluation.

For the purposes of this review, the term KPIs is used as the principal term to describe measurable indicators used to evaluate the delivery and integration of palliative care within ICU settings. For this review, KPIs encompass quality indicators, performance measures and outcome measures reported in the literature. As these related terms are frequently used interchangeably within healthcare quality literature, they will all be incorporated into the search strategy and eligibility criteria to ensure comprehensive identification of relevant evidence. The identified indicators will subsequently be classified according to the Donabedian Model [structure, process, and outcome], ( Donabedian, 1988) to provide a consistent conceptual framework for organising and interpreting the findings.

This study builds on our previous work, which mapped key palliative care characteristics of ICU integration models, by translating these into a preliminary evidence-informed framework of measurable indicators to support system-level evaluation ( King et al., 2026). It supports national research priorities in Ireland by outlining how palliative care delivery can be measured and evaluated for individuals with life-limiting illness in the ICU setting ( All Ireland Institute of Hospice and Palliative Care [AIIHPC], 2025). A scoping review is appropriate for identifying and mapping this diverse evidence base ( Peters et al., 2020). These findings will inform the development of an evidence-based KPI framework to support quality improvement, benchmarking, and accountability in ICU palliative care.

## Protocol

**Study design:** This scoping review will be conducted in accordance with the methodological framework proposed by Arksey and O’Malley (2005), with additional guidance from the Joanna Briggs Institute, and will be reported in line with the PRISMA-ScR guidelines ( Peters et al., 2020).

**Aim**: To identify and map KPIs to inform a framework for evaluating palliative care delivery in ICUs.

### Objectives

• To identify KPIs used to evaluate palliative care delivery in ICUs.
• To map the domains and categories of these indicators.
• To examine how these indicators are defined and measured across studies.
• To inform the development of a preliminary evidence-informed framework that organises domains and KPIs for evaluating palliative care delivery in the ICU

Table 1 details the Population, Concept, and Context (PCC).

**Table 1.** Population, Concept, and Context (PCC) framework guiding the review.

Population	Concept	Context
Adult patients in ICU	KPIs, quality measures & outcome measures	Delivery & integration of palliative care in ICU settings

* ICU = Intensive Care Unit; KPIs = Key Performance Indicators.

**Search strategy:** We will conduct a comprehensive search using controlled vocabulary (e.g., MeSH terms) and free-text terms derived from the PCC framework. The search will focus on three core concepts: palliative care, intensive care and key performance or quality indicators (see Table 2). The full search string for all databases is available on the Open Science Framework (OSF) at 10.17605/OSF.IO/W5J8V. We will combine synonymous terms using ‘OR’ operator within each key concept to maximise search sensitivity. Core concepts will then be combined using the ‘AND’ operator. Truncation and phrase searching will capture variations within terminology. There is no single index term for key performance indicators, so we will use a mix of subject headings and free-text terms. These will include quality indicators, outcomes, audit, benchmarking and improvement. We will search MEDLINE, CINAHL and Embase. See Table 3 for full details. The search will be limited to publications published from 2014 onwards. The date limit is justified by the shift towards integrated palliative care in contemporary acute healthcare policy. This builds on earlier WHO guidance that positioned palliative care alongside active treatment ( WHO, 2002; WHO & Worldwide Hospice Palliative Care Alliance, 2014). Relevant indicators will be identified through systematic searches of bibliographic databases and grey literature sources. Grey literature searches will be conducted by manually searching the websites of the predefined organisations listed in Table 3. Relevant records will be identified using each website’s search function, where available, or by manually browsing relevant sections (e.g., publications, guidelines, policy documents, standards, and reports). Search terms will include combinations of “palliative care”, “intensive care”, “critical care”, “quality indicators”, “key performance indicators” and “outcome measures”. Retrieved records will be screened against the predefined eligibility criteria and duplicate records will be removed before inclusion. The grey literature search process, including the websites searched, search terms used, search dates, and the number of records retrieved, will be documented to ensure transparency and reproducibility. Study selection will be reported using a PRISMA-ScR flow diagram.

**Table 2.** Key concepts used to inform the search strategy.

Concept	Content
#1	Palliative care
#2	ICU/critical care
#3	KPIs/quality indicators

* ICU = Intensive Care Unit; KPIs = Key Performance Indicators.

**Table 3.** Information sources to be searched, including databases and grey literature sources.

Source type	Sources
**Bibliographic databases**	MEDLINE, CINAHL, Embase
**International organisations**	World Health Organisation (WHO), European Association for Palliative Care (EAPC)
**Policy and guideline bodies**	National Institute for Health and Care Excellence (NICE), Health Information and Quality Authority (HIQA), Department of Health (Ireland)
**National health systems**	Health Service Executive (HSE, Ireland)
**Professional organisations**	All Ireland Institute of Hospice and Palliative Care (AIIHPC); Intensive Care Society (UK & Ireland); Society of Intensive Care Medicine (Ireland); American Thoracic Society (USA); Australian and New Zealand Intensive Care Society (ANZICS)
**Research and policy groups**	Center to Advance Palliative Care (CAPC), Robert Wood Johnson Foundation workgroups
**Clinical programmes**	National Clinical Programme for Palliative Care (Ireland)

**Eligibility criteria:** We developed the eligibility criteria using the PCC structure to guide study selection. This strategy will include studies and documents relating to adult patients (≥18 years) receiving care in ICU settings, also known as critical care settings. We plan to exclude studies focusing on paediatric populations. Paediatric palliative care is recognised as a distinct and highly specialised field with different developmental, family-centred models of care and clinical considerations from adult palliative care ( Benini et al., 2022; WHO, 2023). By restricting this review to adult ICU populations, we reduce heterogeneity and ensure that the resulting framework is directly relevant to adult ICU practice. This review focuses on KPIs, quality indicators, and outcome measures relating to the delivery and integration of palliative care within adult ICUs. This includes indicators relating to quality of care, service delivery, clinical processes, patient and family outcomes, audit and benchmarking. Publications that do not explicitly address palliative care as the focus of the intervention, model of care, or evaluation will be excluded. Although some ICU studies examine concepts aligned with palliative care (e.g., family communication or end-of-life decision-making), these concepts may be investigated as part of broader critical care practice rather than as components of palliative care integration. As the aim of this review is to identify KPIs specifically for the delivery and integration of palliative care within ICU settings, the review will be restricted to studies that explicitly address palliative care. This approach maintains the conceptual focus of the review, preserves alignment with the review objectives, and ensures that the identified KPIs are directly relevant to evaluating palliative care integration in adult ICU settings. Eligible grey literature will include policy documents, clinical guidelines, expert consensus statements, standards, organisational reports, and government publications that report or recommend KPIs relevant to palliative care delivery within adult ICU settings. Editorials, opinion pieces, commentaries, conference abstracts, and documents that do not report or describe indicators will be excluded. Limiting the search to publications published from 2014 onwards ensures relevance to contemporary clinical practice and reflects the shift towards integrated palliative care in acute healthcare settings ( WHO, 2014).

**Study selection:** Titles and abstracts will be screened against inclusion criteria, followed by full-text review. Table 4 shows the inclusion/exclusion criteria. Screening will be conducted by two reviewers with discrepancies resolved through discussion with a third arbitrator.

**Table 4.** Inclusion and exclusion criteria for study selection.

Inclusion	Exclusion
Studies and documents describing: • KPIs • quality indicators • outcome measures Related to: • palliative care • ICU/critical care Sources: • peer-reviewed literature • national policy • regulatory frameworks • audit reports • grey literature	• Non-ICU settings • Paediatric populations (<18 years) and paediatric ICU settings • Opinion pieces • Abstracts • Non-peer review sources • Publications published before 2014 • Publications where “palliative care” is not explicitly mentioned

* ICU = Intensive Care Unit

**Data extraction & charting:** We will extract data using a standardised charting form, detailed in Table 5. Planned extraction data include study characteristics (authors, year of publication, country/region, and study design) and ICU type (e.g., medical, surgical, or mixed intensive care units). Planned extracted data will include the type of palliative care delivered (e.g., generalist or specialist modes) and the domains of care (physical, psychological, social, spiritual, and end-of-life). For each identified KPI, we will record name, definition, type (structure, process, or outcome), measurement methods, data sources, and the timing of measurement during an ICU stay.

**Table 5.** Data extraction variables for charting included studies.

Category	Variable	Description/Purpose
**Study Characteristics**	Author(s)	First author and year of publication
	Year of publication (2014-present)	To ensure clinical and policy relevance
	Country/region	To capture geographic context of study
	Study design	e.g. qualitative, quantitative, mixed-methods, policy document
	ICU setting	Type of ICU (e.g. medical, surgical, mixed, specialist ICU)
**Concept**	Type of palliative care delivery	To capture generalist vs specialist palliative care; model of integration within ICU
	Domain of care	Physical, psychological, social, spiritual, end-of-life care
**Indicators/KPIs**	Indicator name	Name/label of KPI or quality indicator
	Indicator definition	To capture how the indicator is defined
	Indicator type	Structure, process, or outcome measure
	Measurement method	To capture how the indicator is measured (e.g. audit, tool, scale)
	Data source	e.g. patient records, ICU databases, administrative data
	Timing of measurement	To capture when indicator is assessed (e.g. ICU admission, during stay, end-of-life)
**Indicator implementation and application**	Purpose of indicator,	To capture why a KPI is used (e.g., Quality improvement, clinical audit, benchmarking, accreditation, performance monitoring, service evaluation, research, quality assurance)
	Level of application	To capture where the KPI is applied (e.g., Patient-level, ICU/unit-level, system-level)
	Implementation considerations	Any reported implementation considerations, including any reported facilitators or challenges to use in ICU,
**Key Findings**	Main findings	To capture main findings relevant to KPI use

* ICU = Intensive Care Unit; KPIs = Key Performance Indicators.

We will also extract information relating to the intended application of each identified KPI, including its purpose (e.g., quality improvement, benchmarking, or audit), level of application (patient, unit, or system level), and any reported implementation considerations. Finally, key findings relevant to the use of KPIs in ICU palliative care will be extracted. These data will support the synthesis, classification, and mapping of KPIs and inform the development of a preliminary evidence-informed framework for evaluating palliative care delivery within adult ICU settings. The data extraction form will be piloted on a sample of included studies and refined iteratively to ensure consistency and relevance. Data will be extracted by one reviewer and independently checked for accuracy by a second reviewer.

**Data synthesis:** The extracted indicators will be synthesised using an inductive descriptive content analysis ( Hsieh & Shannon, 2005). Similar indicators will be grouped into conceptually related categories before being organised into higher-order domains. The resulting domains will be mapped and presented in relation to the review objectives ( Peters et al., 2020), and classified according to the Donabedian Model (structure, process, and outcome) to support interpretation and presentation of the findings ( Donabedian, 1988).

We plan to capture both generalist (primary) palliative care and specialist palliative care concepts, indicators and domains within ICU practice. 

**Results**: The findings will be presented as a descriptive summary of the identified KPIs, including their categorisation into conceptually related categories and higher-order domains. The results will be mapped to the review objectives to provide an overview of the indicators used to evaluate the delivery and integration of palliative care within ICU settings. The identified KPIs will be presented within a preliminary, evidence-informed framework, organised into domains and classified according to the Donabedian Model (structure, process, and outcome). This framework is intended as an organising structure to support interpretation of the findings and inform future framework refinement and validation. It will not represent a formally developed or validated evaluation framework.

Potential limitations of this scoping review will be acknowledged, including restrictions on study design, reporting quality, and the absence of a formal quality appraisal. As this review aims to map KPIs for evaluating palliative care delivery within the ICU, we will include studies regardless of methodological design or quality to ensure comprehensive coverage. This is consistent with guidance from the Joanna Briggs Institute and others, which emphasises that critical appraisal in scoping reviews should be guided by the review objectives ( Arksey & O’Malley, 2005; Peters et al., 2020). Limitations related to the breadth of the search and potential gaps in the available evidence will also be considered.

The findings of this review will inform the development of a preliminary evidence-informed framework for evaluating palliative care delivery in ICUs and identify gaps in the existing evidence base to guide future research and practice.

## Conclusion

This scoping review will map available evidence and policies on KPIs for ICU palliative care. It will clarify these PC KPIs for ICU care and their application. The review will address an important gap in the literature and support the development of a preliminary evidence-informed KPI framework. These findings are expected to inform clinical practice, quality improvement initiatives, and policy development, enhancing the integration and evaluation of palliative care in ICU settings.

## Patient and public involvement

Patients and the public were not involved in the design of this protocol.

## Ethics and consent

Ethical approval is not required as the study involves analysis of publicly available data.

## Dissemination

Findings will be disseminated through peer-reviewed publication, conference presentations, and engagement with policy and clinical stakeholders. The resulting preliminary evidence-informed framework aims to support evaluation and implementation of integrated palliative care within ICU settings.

**Registration**: The protocol has been registered on the Open Science Framework (OSF) ( https://osf.io/w5j8v/).

**Study status:** The study is currently at the development stage.

## Data Availability

No data associated with this article. Open Science Framework (OSF). https://doi.org/10.17605/OSF.IO/W5J8V ( King et al., 2026). Relevant study materials (e.g., collection tools, and supplementary documentation) will be developed during the study and deposited in this file. This project contains the following underlying data:
• Search string • PRISMA-ScR Checklist. Data is available under the terms of the CC-By Attribution 4.0 International.
