# Supplementary material for: Mapping of key performance indicators for palliative care in intensive care settings: A scoping review protocol
Source: HRB Open Res. 2026 Aug 8;9:44. Originally published 2026 Apr 30. [Version 2] doi: 10.12688/hrbopenres.14430.2 (PMC13562920; doi:10.12688/hrbopenres.14430.2)
Supplement: Peer review history

# Reviewer report: Reviewer response for version 2

**DOI:** 10.21956/hrbopenres.16009.r57336

**Published:** 2026-09-10

**Authors:** Sarah Godfrey

**Affiliations:** 1 Mayo Clinic, Rochester, USA

This is a clearly outlined scoping review protocol that addresses an interesting and understudied area of the palliative care literature. It will provide important information to guide clinical practice, quality improvement, and assessment of palliative care interventions. I believe the edits have improved the protocol, and it is now ready for indexing.

Is the study design appropriate for the research question?

Yes

Is the rationale for, and objectives of, the study clearly described?

Yes

Are sufficient details of the methods provided to allow replication by others?

Yes

Are the datasets clearly presented in a useable and accessible format?

Not applicable

Reviewer Expertise:

Palliative cardiology

I confirm that I have read this submission and believe that I have an appropriate level of expertise to confirm that it is of an acceptable scientific standard.

# Reviewer report: Reviewer response for version 2

**DOI:** 10.21956/hrbopenres.16009.r57341

**Published:** 2026-09-08

**Authors:** Yodang Yodang

**Affiliations:** 1 Universitas SembilanBelas November Kolaka, Kolaka Regency, Indonesia

1. The manuscript states that, for the purposes of the review, **KPIs** will serve as an umbrella term encompassing quality indicators, performance measures, and outcome measures. However, these constructs are not necessarily synonymous and may operate at different conceptual and analytical levels. The current operational definition improves clarity but may still lead to conceptual conflation during data extraction and synthesis. For example, an outcome measure may be used as a performance indicator, but not every outcome measure necessarily constitutes a KPI. **Suggestion:** Provide a brief conceptual distinction between these terms and explicitly explain how indicators will be handled when terminology varies across studies. A simple hierarchy or classification approach could improve analytical transparency.
2. There appears to be some methodological ambiguity between the proposed **inductive descriptive content analysis** and the predefined extraction categories. The protocol states that indicators will be grouped inductively into conceptually related categories and higher-order domains. However, Table 5 already specifies domains of care as: physical, psychological, social, spiritual, and end-of-life care. It is therefore unclear whether the domains will be predefined *a priori*; generated inductively from the included evidence; or developed through a combination of deductive and inductive approaches. **Suggestion:**
  Explicitly describe the analytical sequence.
3. The eligibility criteria appropriately include several forms of grey literature, including policy documents, clinical guidelines, expert consensus statements, organisational reports, and government publications. However, Table 4 lists **“Non-peer review sources”** among the exclusion criteria. This creates a direct internal inconsistency because much of the eligible grey literature is, by definition, not peer reviewed. **Suggestion:** Revise this exclusion criterion to specify the types of non-peer-reviewed sources that are excluded.
4. The use of the Donabedian Model provides a useful and established framework for classifying indicators. However, the protocol should provide more detail regarding how classification disagreements will be managed. Some indicators may plausibly fit more than one category. For example, an indicator relating to specialist palliative care availability could have structural and process dimensions. **Suggestion:** specify whether each indicator will be assigned to only one Donabedian category; whether multiple classification will be permitted; how ambiguous indicators will be handled; whether classification decisions will be independently reviewed.

Is the study design appropriate for the research question?

Yes

Is the rationale for, and objectives of, the study clearly described?

Yes

Are sufficient details of the methods provided to allow replication by others?

Partly

Are the datasets clearly presented in a useable and accessible format?

Partly

Reviewer Expertise:

Palliative care, Bereavement care, End-of-life care

I confirm that I have read this submission and believe that I have an appropriate level of expertise to confirm that it is of an acceptable scientific standard, however I have significant reservations, as outlined above.

# Reviewer report: Reviewer response for version 2

**DOI:** 10.21956/hrbopenres.16009.r57116

**Published:** 2026-08-11

**Authors:** Michael Bennett

**Affiliations:** 1 University of Windsor, Windsor, Canada

I have no further comments. I believe the authors have adequately addressed my concerns.

Is the study design appropriate for the research question?

Yes

Is the rationale for, and objectives of, the study clearly described?

Partly

Are sufficient details of the methods provided to allow replication by others?

Partly

Are the datasets clearly presented in a useable and accessible format?

Yes

Reviewer Expertise:

Palliative Care

I confirm that I have read this submission and believe that I have an appropriate level of expertise to confirm that it is of an acceptable scientific standard.

# Reviewer report: Reviewer response for version 1

**DOI:** 10.21956/hrbopenres.15899.r55767

**Published:** 2026-06-22

**Authors:** Jude Ominyi

**Affiliations:** 1 University of Suffolk, Ipswich, UK

- Clarify whether the study is solely a scoping review or also includes formal framework development, and specify the methodology for framework construction if intended.
- Provide clearer operational definitions and distinctions between KPIs, quality indicators, outcome measures, and performance measures to improve conceptual consistency.
- Resolve inconsistencies in date restrictions across the protocol (10 years, 12 years, and from 2014 onward) and provide a single justified timeframe.
- Reconsider excluding studies where “palliative care” is not explicitly stated, as relevant ICU indicators may be described using alternative terminology.
- Justify the use of thematic analysis for KPI synthesis or consider a more appropriate analytic approach for indicator classification and mapping.
- Consider including a stakeholder consultation stage to strengthen the relevance and applicability of the proposed framework.
- Expand reporting of the search strategy, particularly grey literature identification and screening procedures.
- Clarify eligibility decisions regarding policy documents, expert consensus papers, and non-peer-reviewed evidence sources.
- Refine and streamline the data extraction framework to improve feasibility and analytical clarity.
- Address minor language and consistency issues throughout the manuscript.

Is the study design appropriate for the research question?

Yes

Is the rationale for, and objectives of, the study clearly described?

Yes

Are sufficient details of the methods provided to allow replication by others?

Partly

Are the datasets clearly presented in a useable and accessible format?

Partly

Reviewer Expertise:

Palliative care, Implementation science, EBP etc

I confirm that I have read this submission and believe that I have an appropriate level of expertise to confirm that it is of an acceptable scientific standard, however I have significant reservations, as outlined above.

# Response

**Published:** 2026-07-30

**Authors:** Yvonne King

**Affiliations:** School of Nursing & Midwifery, Trinity College Dublin School of Nursing and Midwifery, Dublin, Leinster, Ireland

Dear Dr Jude, Ominyi,

 Thank you for taking the time to review our protocol and for your helpful suggestions. We appreciate your thoughtful and constructive comments, which have helped strengthen the protocol. Please find our responses to your comments below.

**Comment 1** *: “Clarify whether the study is solely a scoping review or also includes formal framework development and specify the methodology for framework construction if intended”.*

**Response**: We have revised the manuscript to clarify that this study is a scoping review and does not include a formal framework development methodology. The primary purpose of the review is to identify, map, and synthesise key performance indicators (KPIs) relating to palliative care delivery in ICU settings. The identified KPIs will be organised into a preliminary, evidence-informed domain-based framework derived from the mapped evidence to support interpretation of the findings and inform future framework refinement and validation. We have also clarified that this represents an organising structure rather than a formally developed or validated evaluation framework. In addition, we specify that the identified KPIs will be classified using the established Donabedian Model (structure, process, and outcome) to support the organisation and interpretation of the finding.

# Response

**Published:** 2026-07-30

**Authors:** Yvonne King

**Affiliations:** School of Nursing & Midwifery, Trinity College Dublin School of Nursing and Midwifery, Dublin, Leinster, Ireland

Dear Dr Jude, Ominyi,

 Thank you for taking the time to review our protocol and for your helpful suggestions. We appreciate your thoughtful and constructive comments, which have helped strengthen the protocol. Please find our responses to your comments below.

**Comment 1** *: “Clarify whether the study is solely a scoping review or also includes formal framework development and specify the methodology for framework construction if intended”.*

**Response**: We have revised the manuscript to clarify that this study is a scoping review and does not include a formal framework development methodology. The primary purpose of the review is to identify, map, and synthesise key performance indicators (KPIs) relating to palliative care delivery in ICU settings. The identified KPIs will be organised into a preliminary, evidence-informed domain-based framework derived from the mapped evidence to support interpretation of the findings and inform future framework refinement and validation. We have also clarified that this represents an organising structure rather than a formally developed or validated evaluation framework. In addition, we specify that the identified KPIs will be classified using the established Donabedian Model (structure, process, and outcome) to support the organisation and interpretation of the finding

**Comment 2:** “ *Provide clearer operational definitions and distinctions between KPIs, quality indicators, outcome measures, and performance measures to improve conceptual consistency.”*

**Response: **We have revised the manuscript to improve conceptual clarity by providing an operational definition for the terminology used throughout the review. Specifically, we have clarified that, for the purposes of this review, the term *KPIs* is used as the principal term to describe measurable indicators used to evaluate the delivery and integration of palliative care within ICU settings. We have also explained that related terms, including quality indicators, performance measures, and outcome measures, are incorporated into the search strategy and eligibility criteria to ensure comprehensive identification of relevant evidence, reflecting the variability in terminology used across the literature. Finally, we have clarified that the identified indicators will be classified according to the Donabedian Model (structure, process, and outcome) (Donabedian, 1988), providing a consistent conceptual framework for organising and interpreting the findings.

 The relevant references have been added to the manuscript to support the revised methodology.

**Comment 3:** “ *Resolve inconsistencies in date restrictions across the protocol (10 years, 12 years, and from 2014 onward) and provide a single justified timeframe.”*

**Response:** We have revised the manuscript to ensure a consistent publication date restriction throughout the protocol. Specifically, we have standardised the eligibility criteria, search strategy, and Table 4 to include publications published from **2014 onwards** and have removed the previous references to "10 years" and "12 years." We have also clarified the rationale for this timeframe, noting that it reflects the shift towards integrated palliative care in contemporary acute healthcare policy following the publication of the World Health Organisation and Worldwide Hospice Palliative Care Alliance guidance in 2014. These revisions improve the clarity, consistency, and reproducibility of the review methodology.

**Comment 4:** *Reconsider excluding studies where "palliative care" is not explicitly stated, as relevant ICU indicators may be described using alternative terminology.*

**Response:** See response to reviewer 1 comment 4

**Comment 5:** “ *Justify the use of thematic analysis for KPI synthesis or consider a more appropriate analytic approach for indicator classification and mapping.”*

**Response:** We agree that the synthesis of KPIs differs from the analysis of qualitative data. We have therefore revised the manuscript to replace thematic analysis with an inductive descriptive content analysis, which is more appropriate for the synthesis and classification of indicators identified through a scoping review. Specifically, extracted indicators will be grouped into conceptually related categories, organised into higher-order domains, and subsequently classified according to the Donabedian Model (structure, process, and outcome). This revision provides a clearer and more methodologically appropriate approach to indicator synthesis, classification, and mapping.

 The relevant references have been added to the manuscript.

**Comment 6:** “ *Consider including a stakeholder consultation stage to strengthen the relevance and applicability of the proposed framework.”*

**Response:** We agree that stakeholder consultation can strengthen the relevance and applicability of evidence syntheses. However, in accordance with current JBI guidance, stakeholder consultation is considered an optional component of scoping review methodology rather than a mandatory stage. As the primary aim of this review is to identify and map the published evidence relating to KPIs for palliative care delivery within ICU settings, we have not included a formal stakeholder consultation stage. We have clarified that the preliminary evidence-informed framework generated through this review is intended to inform future refinement and validation, at which stage stakeholder consultation and consensus methods will be important components of framework development.

**Comment 7:** “ *Expand reporting of the search strategy, particularly grey literature identification and screening procedures.”*

**Response:** We have expanded the Search Strategy section to provide greater detail regarding the identification and screening of grey literature. Specifically, we have clarified that grey literature will be identified through manual searches of predefined organisational websites using each website's search function, where available, and by manually browsing relevant sections (e.g., publications, guidelines, policy documents, standards, and reports). We have also specified the search terms to be used, clarified that all retrieved records will be screened against the predefined eligibility criteria and duplicate records removed before inclusion, and stated that the grey literature search process, including the websites searched, search terms used, search dates, and the number of records retrieved, will be documented to ensure transparency and reproducibility. In addition, we have clarified that study selection will be reported using a PRISMA-ScR flow diagram.

**Comment 8:** “ *Clarify eligibility decisions regarding policy documents, expert consensus papers, and non-peer-reviewed evidence sources.”*

**Response: **We have revised the eligibility criteria to clarify the types of grey literature eligible for inclusion in this review. Specifically, we now state that eligible non-peer-reviewed evidence includes policy documents, clinical guidelines, expert consensus statements, standards, organisational reports, and government publications that report or recommend KPIs relevant to palliative care delivery within adult ICU settings. We have also clarified that editorials, opinion pieces, commentaries, conference abstracts, and documents that do not report or describe relevant indicators will be excluded. These revisions improve the transparency and reproducibility of the eligibility criteria.

**Comment 9:** “ *Refine and streamline the data extraction framework to improve feasibility and analytical clarity.”*

**Response: **We have revised the data extraction framework to improve both feasibility and analytical clarity. Specifically, we have streamlined the extraction variables to focus on information directly relevant to the review objectives, including the characteristics, application, and implementation of identified KPIs. We have also removed redundant and interpretive data items and clarified the purpose of the remaining extraction variables. These revisions ensure that the extracted data directly support the planned synthesis, classification, and mapping of KPIs and the development of a preliminary evidence-informed framework for evaluating palliative care delivery within adult ICU settings.

**Comment 10:** “ *Address minor language and consistency issues throughout the manuscript.”*

**Response: **We have carefully reviewed the manuscript and revised the language throughout to improve clarity, readability, and consistency. Minor grammatical, typographical, and stylistic errors have been corrected, and terminology has been standardised where appropriate to ensure consistency across the manuscript.

# Reviewer report: Reviewer response for version 1

**DOI:** 10.21956/hrbopenres.15899.r55886

**Published:** 2026-06-19

**Authors:** Michael Bennett

**Affiliations:** 1 University of Windsor, Windsor, Canada

This protocol outlines a scoping review that aims to identify and map key performance indicators (KPIs) used to evaluate palliative care delivery within intensive care unit (ICU) settings. The topic is timely and relevant, as the integration of palliative care into ICU practice continues to expand internationally while approaches to evaluating quality and performance remain inconsistent. The rationale, aims, and objectives are clearly stated, and the proposed use of a scoping review methodology is appropriate given the broad and heterogeneous nature of the evidence base.

 Overall, this is a well-developed protocol with a clear focus and a structured methodological approach. I have only a small number of comments that would strengthen the transparency and reproducibility of the review.

 Major Comment:

1. Justification for exclusion of pediatric populations. 

The protocol states that studies focused on pediatric ICU populations will be excluded; however, no rationale is provided for this decision. Because pediatric palliative care differs substantially from adult palliative care in terms of patient populations, family involvement, care trajectories, and quality indicators, I recommend the authors explicitly justify this exclusion and clarify why the review is limited to adult ICU settings.

 Comments for Clarification:

1. Consistency of publication date limits

There appears to be an inconsistency regarding the date restrictions applied to the review. The search strategy states that studies published from 2014 onward will be included, whereas the eligibility criteria table excludes publications older than ten years. These criteria may not be equivalent. Clarification is needed to ensure that the review can be replicated.

1. Grey literature search procedures

Grey literature searches procedures the inclusion of grey literature is a strength of the protocol. However, additional detail regarding how grey literature sources will be searched and selected would improve methodological transparency. For example, it would be helpful to specify how organizational websites will be searched and whether any limits or screening procedures will be applied.

1. Exclusion of studies where palliative care is not explicitly mentioned

The exclusion criteria indicate that publications will be excluded if “palliative care” is not explicitly mentioned. The authors may wish to clarify the rationale for this criterion, as some ICU literature may address concepts closely aligned with palliative care (e.g., end-of-life care, goals-of-care communication, family support, withdrawal-of-treatment decision-making) without using the specific term “palliative care.”

 Overall, the study addresses an important gap in the literature, and the proposed methodology is generally appropriate. Subject to clarification of the points above, I believe the protocol is suitable for indexation.

Is the study design appropriate for the research question?

Yes

Is the rationale for, and objectives of, the study clearly described?

Partly

Are sufficient details of the methods provided to allow replication by others?

Partly

Are the datasets clearly presented in a useable and accessible format?

Yes

Reviewer Expertise:

Palliative Care

I confirm that I have read this submission and believe that I have an appropriate level of expertise to confirm that it is of an acceptable scientific standard, however I have significant reservations, as outlined above.

# Response

**Published:** 2026-07-30

**Authors:** Yvonne King

**Affiliations:** School of Nursing & Midwifery, Trinity College Dublin School of Nursing and Midwifery, Dublin, Leinster, Ireland

Dear Dr Michael Bennett,

 Thank you for taking the time to review our protocol and for all of your helpful suggestions. We appreciate your thoughtful and constructive comments, which have helped strengthen the protocol. Please find our responses to your comments below.

**Comment 1 (Major): *Justification for exclusion of paediatric populations***

**Response:** We have revised the eligibility criteria to provide a clearer justification for restricting the review to adult ICU populations. Specifically, we have clarified that paediatric palliative care is recognised by the World Health Organisation as a distinct and highly specialised field with different developmental, family-centred, and clinical considerations. As the aim of this review is to develop a framework for evaluating palliative care delivery in adult intensive care settings, restricting the review to adult ICUs reduces heterogeneity and ensures that the resulting framework is directly applicable to adult ICU practice. We have also added a supporting reference to the World Health Organisation guidance on paediatric palliative care.

 To note we have also adjusted Table 4, [Inclusion and exclusion criteria for study selection], to  Paediatric populations (<18 years) and paediatric ICU settings to ensure that a mixed adult/paediatric study could not be interpreted as eligible.

**References added:**

- Benini, F., Papadatou, D., Bernada, M., Craig, F., De Zen, L., Downing, J., Drake, R., Friedrichsdorf, S., Garros, D., Giacomelli, L., Lacerda, A., Lazzarin, P., Marceglia, S., Marston, J., Muckaden, M. A., Papa, S., Parravicini, E., Pellegatta, F., & Wolfe, J. (2022). International Standards for Pediatric Palliative Care: From IMPaCCT to GO-PPaCS. *Journal of Pain and Symptom Management, 63*(5), e529–e542.
- World Health Organisation. (2023, June 1). *Palliative care for children*. https://www.who.int/europe/news-room/fact-sheets/item/palliative-care-for-children accessed on 26/06/2026

**Comments for Clarification:**

**Comment 2:** ***Consistency of publication date limits***

**Response**: We have revised the manuscript to ensure consistency in the application and reporting of the publication date limits throughout the protocol. Specifically, we have standardised the eligibility criteria to include publications published from 2014 onwards and have amended the exclusion criteria in Table 4 to state " *Publications published before 2014*," thereby aligning the eligibility criteria with the search strategy. We have also revised the accompanying text to consistently describe the rationale for selecting publications from 2014 onwards. These revisions improve the clarity, consistency, and reproducibility of the review methodology.

**Comment 3:** ***Grey literature search procedures***

**Response:** We have expanded the Search Strategy section to provide additional detail regarding the grey literature search procedures. Specifically, we have clarified that grey literature will be identified through manual searches of the predefined organisational websites listed in Table 3, using each website's search function where available and by browsing relevant sections (e.g., guidelines, policy documents, standards, and reports). We have also specified the search terms to be used, stated that all identified documents will be screened against the predefined eligibility criteria, and indicated that the search process (including websites searched, search terms, search dates, and records identified) will be documented to improve the transparency and reproducibility of the review.

**Comment 4: *Exclusion of studies where palliative care is not explicitly mentioned***

**Response: **We agree that some ICU studies examine concepts aligned with palliative care, such as family communication or end-of-life decision-making, without explicitly using the term "palliative care." To address this, we have revised the eligibility criteria to clarify the rationale for this exclusion criterion. Specifically, we have explained that studies not explicitly addressing palliative care as the focus of the intervention, model of care, or evaluation will be excluded, as these concepts may be investigated as part of broader critical care practice rather than as components of palliative care integration. This clarification ensures that the review remains conceptually focused on identifying key performance indicators for the delivery and integration of palliative care within adult ICU settings, while maintaining alignment with the review objectives

 References added

 Benini, F., Papadatou, D., Bernada, M., Craig, F., De Zen, L., Downing, J., Drake, R., Friedrichsdorf, S., Garros, D., Giacomelli, L., Lacerda, A., Lazzarin, P., Marceglia, S., Marston, J., Muckaden, M. A., Papa, S., Parravicini, E., Pellegatta, F., & Wolfe, J. (2022). International Standards for Pediatric Palliative Care: From IMPaCCT to GO-PPaCS. *Journal of Pain and Symptom Management, 63*(5), e529–e542.

 World Health Organization. (2023, June 1). *Palliative care for children*. https://www.who.int/europe/news-room/fact-sheets/item/palliative-care-for-children accessed on 26/06/2026
